# Mini review about metal organic framework (MOF)-based wearable sensors: Challenges and prospects

**DOI:** 10.1016/j.heliyon.2023.e21621

**Published:** 2023-10-26

**Authors:** Hicham Meskher, Samir Brahim Belhaouari, Fariborz Sharifianjazi

**Affiliations:** a Division of Process Engineering, College of Science and Technology, Chadli Bendjedid University, 36000, Algeria; b Division of Information and Computing Technology, College of Science and Engineering, Hamad Bin Khalifa,Doha, Qatar; c School of Science and Technology, The University of Georgia, Tbilisi, Georgia

**Keywords:** flexible materials, Health monitoring applications, iontophoresis, metal organic framework, wearable sensor

## Abstract

Among many types of wearable sensors, MOFs-based wearable sensors have recently been explored in both commercialization and research. There has been much effort in various aspects of the development of MOF-based wearable sensors including but not limited to miniaturization, size control, safety, improvements in conformal and flexible features, improvements in the analytical performance and long-term storage of these devices. Recent progress in the design and deployment of MOFs-based wearable sensors are covered in this paper, as are the remaining obstacles and prospects. This work also highlights the enormous potential for synergistic effects of MOFs used in combination with other nanomaterials for healthcare applications and raise attention toward the economic aspect and market diffusion of MOFs-based wearable sensors.

## Highlights

• Review article on metal organic framework (MOFs)-bsed wearable sensors.
• The advantages of using MOFs as sensing platform and their healthcare applications are discussed.
• The use of MOFs combinations with other materials in the production of electrochemical sensors is also discussed.
• MOF-basedsensors manufacturing, achievements, market diffusion and related limitations are described in depth.
• Challenges and prospects using MOFs-based wearable sensors have been proposed.

## Graphical abstract

**Figure:**
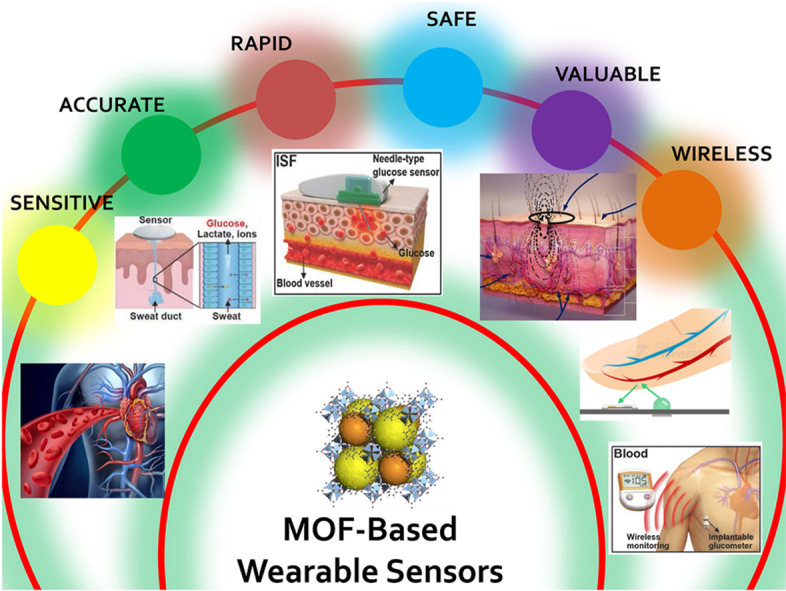

## 1 Introduction

Wearable sensors have recently gained a lot of interest due to their exceptional performances and numerous benefits. The market demand for these technologies has rapidly expanded from $20 billion in 2016 to $80 billion in 2022 [1] because of their real-world applications that require quick, accurate detections. They have been utilized for a wide range of tasks including virus detection, cancer early diagnosis and monitoring, and pulse rate analysis [[2], [3], [4], [5], [6]].

In addition to sensing of various physiological variations, wearable sensors should be able to detect biomarkers specific to diseases at very early stages of their emergence, then it can help accurate diagnosis of diseases like DNA damage [7], Alzheimer's [8], and cancer [9,10]. For this purpose, numerous various nanomaterials have been developed as sensing elements since the sensing matrix is the most crucial component of any sensors. There has been much improvement in the sensitivity and selectivity of the sensors. the most commonly used nanomaterials for sensing purposes include nanostructures like carbon-based materials [11,12], MXenes [13], conducting polymers like molecularly imprinted polymers (MIPs) [14], metals and metal oxides, and most recently metal organic frameworks (MOFs) [[15], [16], [17], [18]].

Metal-organic frameworks (MOFs), commonly referred as porous coordination polymers (PCPs), are created when metal ions or clusters and organic bridging ligands are coordinated [19,20]. Extended infinite networks are created by the coordination of metal ions/clusters with ligands [21]. So far, various metal ions and organic ligands have been explored. As a result, a variety of MOF types haven been demonstrated with outstanding properties [[22], [23], [24]]. MOFs have wide structural variety and tunable physicochemical features since there are so many possible metal and ligand combinations. MOFs are benefited from the inherent advantages of both flexible organic materials and hard inorganic elements. Moreover, MOFs' distinct architectures can result in extremely high Langmuir surface area (>10,000 m^2^ g^−1^) [25]. Hence, MOFs have been used for desalination, water treatment, and pollution remediation due to their adjustable pores and high porosity [19,26,27], energy related applications [28], catalysis, and sensing applications [20,29,30]. Furthermore, MOFs can be frequently coupled with other nanostructures to create effective MOF-based hybrids for the development of sensors and biosensors because of their physicochemical alterations and tunable chemical functionalization [[21], [31]]. For example, Recently, SARS-CoV-2 and other viral diseases have been detected using MOF-based sensors [32,33].

This review aims to present the most recent uses of MOFs in wearable sensors for biomedical applications. While illustrative examples of these sensors are presented, basic architectures and operating principles of wearable sensors based on MOFs are reviewed. The key features of using MOFs as sensing platforms are highlighted. In addition, an outlook about the opportunities of these materials in the field of sensing is also provided along with an overview of the current challenges in the development of MOF-based wearable sensors for glucose detection as an example.

## 2 Research procedure

**Scheme 1:**
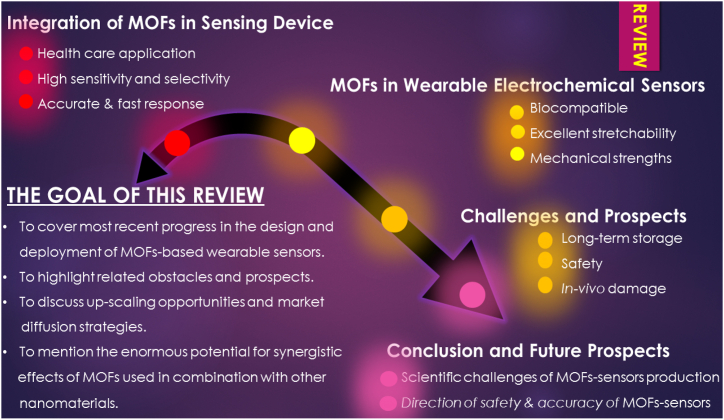
Research strategy and review over flow.

We conducted a Google Scholar search to identify references containing information on the MOFs, electrochemical (bio)sensing, wearable sensors, health diagnosis, commercialization and economic aspect of MOFs-based wearable sensors. We used single or combinatorial searches on keywords such as “MOFs”, “commercialization”, “health diagnosis”, “economic aspect”, “safety”, “glucose”, “biocompatibility”, etc. For each identified paper, we then reviewed the references therein to determine if additional relevant references could be identified. The initial search revealed more than 150 papers, from which 130 were selected for consideration in the present review to cover most recent progress in the design and deployment of MOFs-based wearable sensors are covered in this paper, as are the remaining obstacles and prospects. This work also highlights the enormous potential for synergistic effects of MOFs used in combination with other nanomaterials for healthcare applications and raise attention toward the economic aspect and market diffusion of MOFs-based wearable sensors (Scheme 1).

## 3 Integration of MOFs in sensing device

The sensing matrix should be integrated into the working electrode of the sensor for electrochemical sensor applications (Table 1), and it should be exceptionally sensitive to the target analyte [17,34,35]. A wide range of processes, including printing, electrodeposition, laser ablation, and drop-casting, can be used for integrating the sensing matrix into the electrode. As a promising sensing matrix, MOFs have gained huge interest for a variety of sensing devices.

**Table 1:** Most recent wearable sensor based on MOFs for health-care applications.

Article title	Authors and Publication date	MOF	Application and Analytical Performance	Outcomes
Design and fabrication of novel flexible sensor based on 2D Ni-MOF nanosheets as a preliminary step toward wearable sensor for onsite Ni (II) ions detection in biological and environmental samples.	Elashery et al., 2022 [18]	Ni-MOF Metal: Ni Ligand: benzenedicarboxylic acid: (BDC))	Determination of Nickel ions in biological and environmental samples. LR = 1.0 × 10^−5^–1.0 × 10^−1^ mol L^−1^. LOD = 2.7 × 10^−6^ mol L^−1^.	• The prepared sensor exhibited excellent performance and fast response to Nickel ions detection. • A lower LOD around 2.7 μM has been obtained. • The sensor exhibited great recoveries in real samples such water human saliva, sweat and tap water
Highly Stretchable Wearable Electrochemical Sensor Based on Ni–Co MOF Nanosheet-Decorated Ag/rGO/PU Fiber for Continuous Sweat Glucose Detection	Shu et al., 2021 [23]	Ni–Co-MOF Metal: Ni–Co. Ligand: benzenedicarboxylic acid: (BDC))	Detection of glucose in sweat Sensitivity = 425.9 μA mM^−1^·cm^−2^. LR: 10 μM–0.66 mM.	• The fabricated electrode exhibited excellent electrochemical performance with high stretchability. • The presence of rGO in the sensing matrix has significantly improved the performance of the (Ni–Co MOF/Ag/rGO/PU) toward glucose detection with a sensitivity around 425.9 μA mM^−1^·cm^−2^. • The wearable sensor exhibited outstanding mechanical flexibility and stretching.
MOF-derived porous Ni/C material for high-performance hybrid nanogenerator and self-powered wearable sensor	Gui et al., 2023 [36]	Ni-MOF Metal: Ni–Co Ligand: Terephthalic acid	Motion monitoring	• A hybrid nanogenerator integrated by a two-layer zigzag triboelectric nanogenerator and an EMG, has been fabricated. • The sensor harvested the mechanical energy generated from human walking for self-powered personnel positioning. • The effect of the TENG structure on the output is analyzed and showed potential for hand motion and gait monitoring.
Fabrication of a sensitive and fast response electrochemical glucose sensing platform based on co-based metal-organic frameworks obtained from rapid in situ conversion of electrodeposited cobalt hydroxide intermediates	Shahrokhian et al., 2020 [37]	Co3 (BTC) 2 MOFs Metal: Co Ligand: 1,3,5-benzene tricarboxylic acid (H3 BTC, C9H 6O 6)))	Glucose detection in human blood LR = 1 μM - 0.33 mM LOD = 0.33 μM.	• A three steps cost-effective and eco-friendly synthesis procedure has been successfully proposed to synthesis crystalline Co 3(BTC) 2 MOFs. • The sensing matrix exhibited excellent performance toward glucose detection and provided outstanding analytical parameters.
Smartphone light-driven zinc porphyrinic MOF nanosheets-based enzyme-free wearable photoelectrochemical sensor for continuous sweat vitamin C detection	Yan et al., 2023 [38]	Zn-MOF Metal: Zn Ligand: 4,4′-biphenyldicarboxylic acid (BPDC))	Detection of vitamin C in human sweat. LR = 10–1100 Μm. LOD = 3.61 μM.	• A smartphone-connected enzymatic sensor to detect vitamin C in human sweat has been successfully developed. • The sensor exhibited great potentianl to ensure proper nutritional balance. • The non'enzymatic sensing platform is constructed by a two-dimensional zinc porphyrinic MOF nanosheets/multi-walled carbon nanotubes (2D-TCPP(Zn)/MCNTs).
A highly flexible Ni–Co MOF nanosheet coated Au/PDMS film based wearable electrochemical sensor for continuous human sweat glucose monitoring	Shu et al., 2018 [17]	Ni–Co-MOF Metal: Ni–Co Ligand: Terephthalic acid (PTA))	Glucose Detection in sweat LR = 20 μM–790 μM. Sensitivity = 205.1 μA mM^−1^ cm^−2^.	• A stretchable sensor based on Ni–Co-MOF is prepared. • Operational paramaters such as Ni: Co ratios were optimized. • The sensor exhibited excellent performance once it is attached to the skin to effectively detect glucose in sweat. • The sensing matrix exhibited excellent performance toward glucose detection and provided outstanding analytical parameters with a high sensitivity of 205.1 μA mM−1 cm−2.
Fluorescent wearable platform for sweat Cl ‐ analysis and logic smart‐device fabrication based on color adjustable lanthanide MOFs	Xu et al., 2013 [35]	DUT‐101 was synthesized by using biphenyl‐4,4′‐dicarboxylic acid (H 2bpdc) with Tb(NO 3) 3 ∙6H 2 O	Cl^−^ ions detection in sweat LOD = 0.1 mM	• A fluorescence wearable sensor to detect Cl^−^ ions in human sweat is proposed. • The sensor exhibited lower limits of detection and quantification with high sensitivity and excellent selectivity to ward Cl‐ ions. • This system is a simple and effective solution for wearable sweat‐based monitoring.
Chiral MOF Derived Wearable Logic Sensor for Intuitive Discrimination of Physiologically Active Enantiomer	Yang et al., 2023 [39]	Chiral *γ*-cyclodextrin metal-organic framework (CDMOF)	Lactate enantiomers	• A dual responsive chiral sensor RT@CDMOF through in situ self-assembly of chiral *γ*-cyclodextrin MOF was prepared. • The embedded RGH and TCN inherit the chirality of host CDMOF, producing dual changes both in fluorescence and reflectance. • A flexible membrane sensor is successfully fabricated based on RT@CDMOF for wearable health monitoring. • Based on above, a chiral implication logic unit can be successfully achieved, demonstrating the promising potential of RT@CDMOF in design and assembly of novel smart devices.
Ultra-thin 2D bimetallic MOF nanosheets for highly sensitive and stable detection of glucose in sweat for dancer	Mao et al., 2023 [27]	The NiMn-MOF Metal: Ni–Mn Ligand: 1,3,5 Benzenetricarboxylic acid (H_3_BTC)	Glucose Detection in sweat Sensitivity = 1576 μA mM^−1^ cm^−2^. LR = 0–0.205 mM. LOD, 0.28 μM.	• Accurate, fast response and sensitive wearable sensor during dancing is successfully fabricated. • The sensing matrix is based on a bimetallic NiMn-MOF and exhibited excellent catalytic activity. • The ultrathin nanosheet and heterogeneous metal ions in the structure optimize the electronic structure, which improves the electrical conductivity of MOFs. • The sensing matrix exhibited excellent performance toward glucose detection and provided outstanding analytical parameters with a high sensitivity of 1576 μA mM^−1^ cm^−2^.
A wearable sweat electrochemical aptasensor based on the Ni–Co MOF nanosheet-decorated CNTs/PU film for monitoring of stress biomarker	Su et al., 2023 [24]	Ni–Co-MOF Metal: Ni–Co Ligand: diamino terephthalic acid (C_8_H_6_O_4_),	Cortisol detection LR = 0.1–100 ng/mL. LOD = 0.032 ng/mL.	• A three steps cost-effective and eco-friendly synthesis procedure has been successfully proposed to synthesis MOF/CCP. • The sensor exhibited excellent analytical performance toward cortisol with high sensitivity and with high repeatability and good selectivity. • The sensor exhibited promising potential for quantitative stress monitoring and management. • The fabricated sensor provided outstanding sensitivity due to the formation of the aptamer-cortisol complex.

The particular structure of MOFs helps enhance the sensors' sensitivity and selectivity, both of which are regarded as essential characteristics of any sensors. For instance, an MOF nanostructure that contains manganese has been solvothermally created. With a detection limit estimated to be around 0.12 ppb, the synthesized nanohybrid demonstrated excellent water-stability and high affinity of nitrogen atoms on the frameworks to Cd^2+^. This indicates the excellent selectivity and anti-interference capacity of the synthesized MOF based composite [40]. Shahrokhian et al. [37], reported a quick and simple three-step synthesis process to coat the GCE active area by Co3(BTC)2 MOFs to produce a sensitive and selective matrix for glucose identification in human blood. The modified electrode showed two wide linear ranges of 1–0.33 mM and 0.33–1.38 mM with high sensitivity of 1792 mA/cm2 and 1002 mA/cm2, respectively [37]. However, it is necessary to always optimize the effects of the electrolyte content, current density, and conductive salt on the synthesis of MOF films [41].

On the other hand, Naghian et al. [42], revealed a straightforward electrochemical detection technique based on the modification of a screen-printed carbon electrode (SPCE) by drop-casting a metal-organic framework (MOF) with extremely high porosity, a sizable surface area, and good thermal and chemical durability on its surface. The suggested method assisted in creating a selective, sensitive, and cost-effective sensor that performed well on actual samples like blood and urine [42]. Similarly, Fernandez and coauthors made an effort to investigate the performance of 2D printers to produce composites made of ionic liquid (IL) and MOF that function as thin film sensors. In order to achieve this, the MOF is solvothermally produced and then impregnated with the IL. The IL/MOF systems are then tested on a specially designed gas flow equipment after being sprayed into a 2D manufactured silver capacitive circuit [43]. These materials provide infinite options for improving the prepared sensors’ analytical performance to detect the intended analyte and expand the field of wearable sensors. MOFs can be synthesized via laser ablation for use in sensing applications. In a fantastic work, a revolutionary method for creating MOF composites (Eu2O3@[Zn2(1,4-ndc)2dabco) by pulsed laser ablation in flowing liquid) was described. The composite Eu2O3@[Zn2(1,4-ndc)2dabco] has a BET specific surface area of 1087 m2/g, and physical and chemical analysis of the produced MOF revealed that the nanoparticles of the framework have an average size of 3.08 nm and exhibited well designed and uniform distribution throughout the crystal [44].

## 4 MOFs in wearable electrochemical sensors

Due to their advantages, such as quick and accurate detection, wearable sensors have recently gained a lot of attention. Indeed, as shown in Table 2, several objectives have been achieved but several issues need to be addressed in the nearest future to improve the applicability of MOFs-based wearable sensors. They also show tremendous promise for use in health care applications, such as the diagnosis and monitoring of diseases [45]. However, due to the majority of wearable sensors that are currently in use primarily recording physical activity and/or vital signs, their usage in healthcare is still limited. Therefore, several attempts must be made to resolve this issue. Wearable biosensors have been extensively used in numerous studies recently to examine biochemical and biological analytes in order to broaden the uses of such devices in human health diagnostics [46,47]. Even though practically all clinical tests now involve human blood, blood collection is still unpleasant and might spread diseases if the equipment is not properly cleaned [48]. On the other hand, sample storage is still a difficult problem that restricts long-term and continuous monitoring as well as transportation. In order to avoid problems with blood samples and to assure long-term accurate health diagnosis, future wearable sensors should be designed to analyze sweat, tears, saliva, or even sweat samples.

**Table 2:** Most recent advances in MOFs-based wearable sensors and related limitations.

MOFs-based wearable sensors	Achievements	Obstacles and Limitations
	• Outstanding porous surface. • Ordered and controllable structure. • Easily miniaturized. • Ease of functionalization. • Excellent biocompatibility. • Flexible and very applicable for motion wearable sensors. • High strechability and high physicochemical resistance	• Production costs. • Upscaling and market diffusion related issues. • *In-vivo* damage. • Real sample complications. • Pretreatment and calibration. • Long-term storage. • Safety and biodegradability.

### 4.1 Properties of the MOF-based wearable sensors

Given its unique properties, MOFs may be seen in this context as an emerging material for sensitive wearable sensors and biosensors [17,49]. The following are the reasons why MOFs should be employed in wearable biosensors. First of all, some MOFs have electrocatalytic activity toward the water-splitting reaction [50], which enhances the detection selectivity because they don't reduce energy consumption only, but MOFs can also determine the reaction pathway in a significant way. Second, several metal-MOF combinations exhibit remarkable electrocatalytic activity that is even competitive with those of these metals [51]. Third, nanomaterials that can have a synergistic influence on the sensing reaction can be accommodated on the MOF using it as a scaffold [52].

### 4.2 Insights on the recent applications of MOFs and conductive materials

MOFs have great porosity, flexibility, and surface area ratio, but their low conductivity makes it difficult to use them in sensing applications. Regarding this, numerous works have developed a variety of pairings of MOFs with more conductive materials like metals and carbon-based materials like graphite, reduced graphene oxide, and CNTs, including both SWCNTs and MWCNTs. Such combinations increase the conductivity and flexibility of MOFs, making them potentially effective composite materials for flexible electronics and bioelectronics [[53], [54], [55]].

**Fig. 1:**
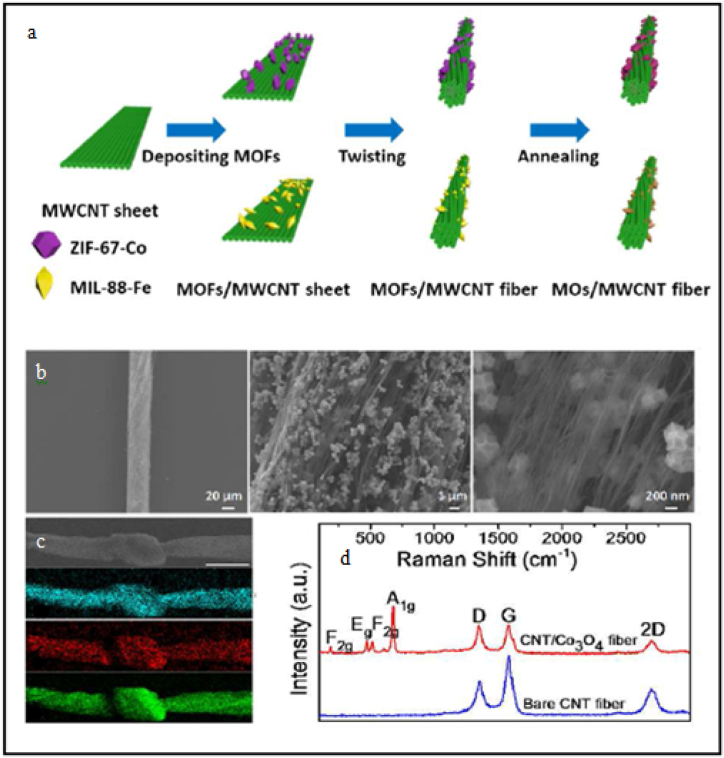
Synthesis of MOFs based composites (a) and their characterization using SEM (b), mapping (c) and Raman spectra (d). Reproduced with permission from ACS [56].

An excellent study in this context described a unique, flexible, wearable device made of highly conductive MWCNTs and MOFs (ZIF-67-Co and MIL-88-Fe). According to the study, after being coupled with MWCNTs to form organic-inorganic fibers, the synthesized MOFs were properly post-treated. The synthesized materials are expected great potential in electrochemical sensing and also in energy storage applications due to their high surface area, presence of Co/Fe elements, and unique structural features (such as dodecahedral morphology). As shown in Fig. 1, MWCNT sheets that were aligned and combined with the MOFs (Fig. 1a) had controlled dimensions were taken from the arrays and placed one on top of the other as can be seen in Fig. 1b–d. Pre-fabricated MOFs, such as ZIF-67-Co and MIL-88-Fe, were injected into the stacked MWCNT sheets using an easy drop-casting technique as a proof of concept. The required amount of MOFs was tweaked by adjusting the volume of the MOFs/ethanol suspension [56].

**Fig. 2:**
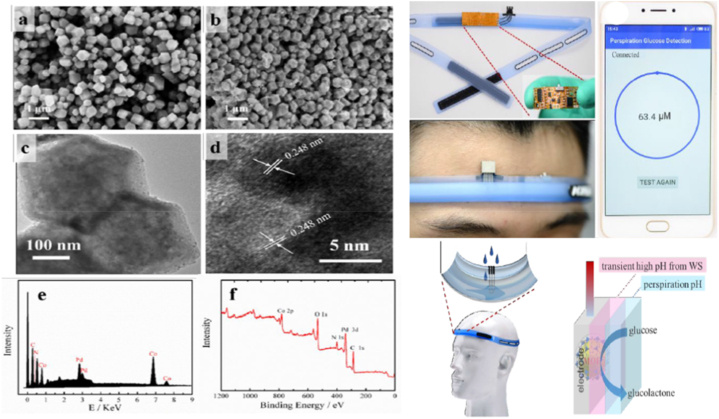
Palladium-based metal organic framework (Pd@ZIF-67)-based sweat glucose wearable sensor. Scanning electron micrographs of (a) ZIF-67 and (b) Pd@ ZIF-67. (c) Transmission electron micrographs of Pd@ZIF-67. (d) High resolution transmission electron micrographs of the Pd NPs in Pd@ZIF-67. (e) Energy-dispersive spectrum of Pd@ZIF-67. (f) X-ray photoelectron spectrum of Pd@ZIF-67.Reproduced with permission from ACS [57].

Similarly, A palladium-based metal organic framework (Pd@ZIF-67)-based sweat glucose wearable sensor has been created. The synthesized Pd@ZIF-67 was processed using a water splitting reaction to preserve the required alkaline environment for efficiently detecting glucose. During the pretreatment procedure, Pd worked as a reducing agent of H_2_, ensuring a stable and selective detection platform. For wearable perspiration sensing, the wearable sensor was attached to a sweatband. With analytical results remaining consistent for two months, the proposed Pd@ZIF-67 could be a viable long-term solution for sensitive and stable glucose detection in future non-invasive, painless, and practical glucose tests for self-monitoring of diabetes (Fig. 2) [57]. SEM analysis revealed that ZIF-67 had a polyhedral structure with a relatively smooth surface, and Pd@ZIF-67 exhibited a similar polyhedral structure but with a relatively rough surface (Fig. 2a and b) while the TEM image (Fig. 2c) shows that the Pd NPs were encapsulated in ZIF-67. More detailed structure information about the Pd NPs can be found in the HRTEM image (Fig. 2d). From the image, the lattice spacing of fringes on the Pd NP was found to be about 0.248 nm, which indicates face-centered cubic Pd facets (0.246 nm) and that the Pd NPs were grown along the (111) facet direction.38 Characteristic peaks of C, N, Co, and Pd were observed from the EDS of the Pd@ZIF-67 particle (Fig. 2e). The valence state of each element in the Pd@ZIF-67 NPs was examined by XPS as shown in Fig. 2f.

### 4.3 MOF-based self-powered flexible piezoelectric sensors

**Fig. 3:**
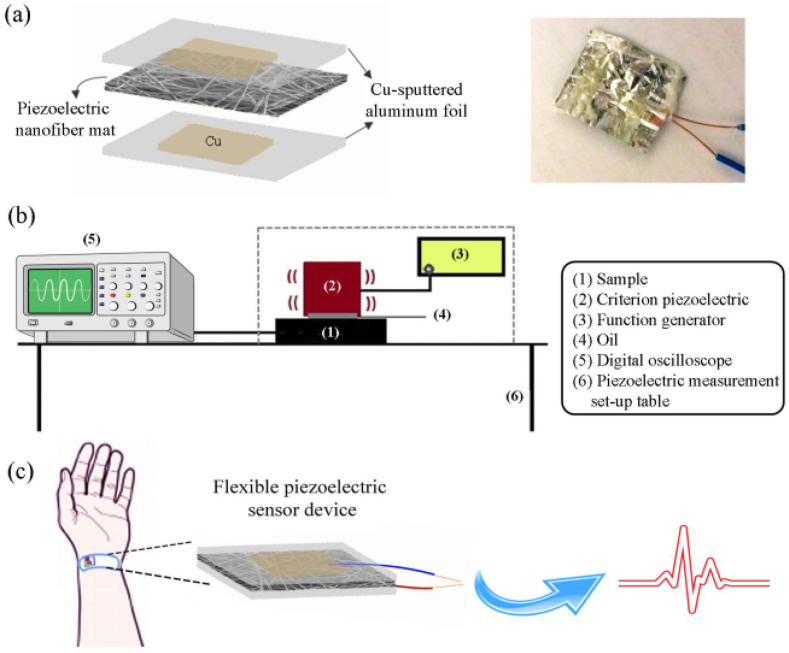
(a) Flexible sensor architecture, (b) measurement set up, and (c) pulse signals recording using the prepared sensor. Reproduced with permission from ACS [58].

It is urgent to use self-powered flexible piezoelectric sensors since the old bioanalytical tools systems have numerous shortcomings caused by their inflexible shape and high power consumption. A novel flexible wrist pulse sensor has been reported in this regard [58]. The MOFs employed (UiO-66 nanoparticles) were made using a solvothermal procedure to give them a more crystalline nature. The nanoparticles made were then electrospun at the same time as the Nafion PVDF solution. This leads to develop flexible membranes with a high output voltage and excellent sensitivity (Fig. 3). The fabricated sensor was produced using a PVDF nanofiber combined with the MOF NPs and Cu-sputtered aluminum foil (Fig. 3a). For measuring (Fig. 3b), a criterion piezoelectric, function generator, and an oscilloscope was used to construct the experimental setup for the *in-situ* detection of radial artery pulse signals (Fig. 3c).

Combining MOFs with metals offers up new possibilities for MOFs in applications other than sensing. Due to ZnO's direct bandgap wide-bandgap semiconductor's superior optical and electrical properties, MOFs are expected to join with ZnO to produce type-II heterojunctions, which is helpful for effective charge separation. For instance, by layer-by-layer (LbL) growing Cu3(HHTP)2 on the surface of ZnO thin film, a novel composite based on ZnO and MOFs (Cu3(HHTP)2) has been generated to prepare type-II heterojunction UV photodetector [59]. The MOF layer's development cycles, variations in the power density of the light source, and light incidence direction were all considered when analyzing photodetection performance. The sensor exhibits 78.2 A/W response and 3.8109 Jones detectivity at 1 V. Our MOF device is therefore highly resistant to bending and fatigue, making it a good candidate for wearable photodetection [59].

### 4.4 Advances in MOFs sensitivity

Additionally, in the past 10 years, efforts have been made to increase the sensitivity of wearable pressure sensors so they can be used in a wider range of medical applications. In order to achieve fast and accurate health monitoring, wearable pressure sensors must be produced with high sensitivity, wide sensing range, excellent reproducibility and/or reusability, and with few restrictions and drawbacks related to small mechanical deformations such as physical motions of fingers. Recently, various skin wearable sensors have b een developed, however, often limited by air and moisture, which restrict their long-term applicability and make it too difficult to obtain tight adhesiveness to the skin [60,61].

**Fig. 4:**
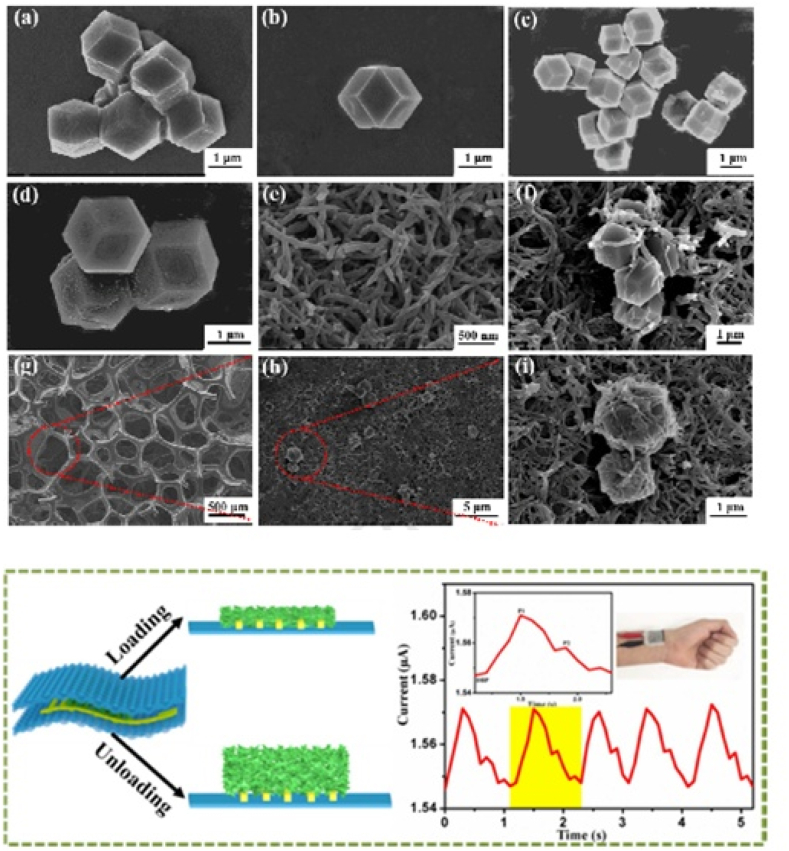
Sandwiched C-MOF/PANIF@PU based wearable pressure sensor. (a–b) SEM image of the ZIF-8, (c–d) SEM image of C-MOF, (e) SEM image of the PANIF, (f) SEM image of the C-MOF/PANIF, (g–i) SEM images of the C-MOF/PANIF@PU. Reproduced with permission from Elsevier [62].

In this regard, a sandwiched C-MOF/PANIF@PU-based wearable pressure sensor has been fabricated (Fig. 4). C-MOF and PANIF are combined to create interconnected nanocomposites, which are then dipcoated onto PU sponges to create C-MOF/PANIF@PU with high specific surface areas, porous microstructures permeable to air and liquid, and reliable elasticity. The rhombic dodecahedron ZIF-8 crystals were synthesized in methanol solution (Fig. 4a and b), which were carbonized at 800 °C for 4 h to obtain the relatively stable C-MOFs (Fig. 4c and d) with high specific surface area, good electrical conductivity and mechanical stability. After mixing with PANIF (Fig. 4e), the interconnected nanocomposites of C-MOF/PANIF were obtained (Fig. 4f). After combination, the prepared conductive C-MOF/PANIF@ PU (Fig. 4g–i) had good compressibility and bend-ability [62]. The prepared sensor exhibited a wide range up to 60 kPa and provided excellent sensitivity and fast response, stable breathability, wireless biomonitoring, and a remarkable repeatability over 15000 cycles. The sensor can also be used to track both small and large human movements, including blood pressure and cheek occlusion (finger bending, and finger pressing) [62].

Unfortunately, there aren't many research reporting MOF-based wearable sweat sensors in the literature so far. However, a number of research that were published in this field and offered some answers that ought to be taken into account in the near future to enhance MOFs' potential future utility in creating more precise wearable sensors. Sweat is a readily available, non-invasive fluid that can be drawn from a person's body during any physical activity, as well as on demand using thermal heating or a chemical induction process known as iontophoresis [63,64].

Sweat analysis is more advantageous than blood diagnosis. However, this method has a number of issues and restrictions that make it difficult to create wearable sweat sensors based on MOF. Four significant issues need to be resolved: (i) the inability to control sweat production rates; (ii) the impact of health circumstances, such as pregnancy, on sweat production; (iii) the limitations of iontophoresis; and (iv) the stretchable wearable heater that makes diagnosis uncomfortable and annoying. By regulating sweat generation and enhancing its storage and transportation, efforts should be made to develop MOF-based wearable sweat sensors [65].

For instance, because patients frequently detest such non-invasive approaches, producing perspiration through physical activity is not a desirable technique. Since excessive sweating makes it uncomfortable to take showers six times a day for a diabetic person. Accurate glucose detection requires diabetic patients to produce sweat during six times each day from breakfast to dinner [3,66]. Even a healthy individual won't ever feel at ease with such a diagnosis and won't be able to withstand six times as much physical activity to produce enough sweat to ensure continues glucose monitoring during the whole day.

Additionally, once perspiration starts with any physical activity, glucose concentrations decrease over time in sweat because it is quickly ejected from the sweat gland. The diluting effect brought on by an increase in sweat rate during an extensive exercise, is thought to be responsible for the lower levels of sweat glucose [67,68]. In addition to the increase in perspiration rate that causes glucose dilution during exercise, the rise in skin temperature will also have an impact on the activity of glucose oxidase, which must be taken into consideration to prevent overestimating the exact glucose concentration [69].

Despite similar patterns, different body areas sweat at different rates, which, as a result of the dilution effect, because varying glucose concentrations at any given time.

**Fig. 5:**
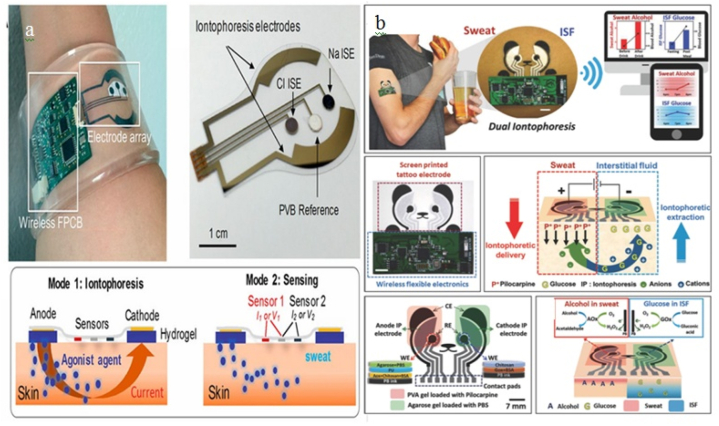
(a)Wearable sweat sensor for glucose detection. Reproduced with permission from PNAS [70]. (b)Wearable sweat sensor for sweat (iontophoresis) detection Reproduced with permission from Wiley [71].

In conclusion, it is highly unlikely that sweat glucose monitoring that is accurate, real-time, continuous, and achieved through exercise sweat will be the best course of action, especially for diabetic persons who suffer from other serious diseases such as blood pressure and heart related disease (Fig. 5a and b). Furthermore, this strategy probably won't be the best for people who are aged, disabled, or pregnant.

Iontophoresis is a well-known technique for health monitoring and therapeutic medicines, particularly for sedentary individuals [72]. It works by administering a modest electrical current to each electrode to polarize electrode's surface. In the iontophoresis process, a sweat-inducing substance is iontophorized into the skin (sweat glands) to produce perspiration [73]. As a result, sweat can be created on-demand and without invasion at any convenient area on the body.

To collect local sweat effectively, the system should have embedded iontophoresis capabilities. The sensing area is sealed to collect sweat using an iontophoresis technique with the sweat-rate sensor mounted on the positive electrode [70]. Additionally, constant secretion rates greater than 100 nL/min/cm^2^ may be reached by carefully constructing the iontophoresis electrodes and improve sweat stimulation of the medication in the synthesized films. It was possible to extract enough perspiration for an accurate in-situ glucose analysis without endangering the individuals' epidermis or making them uncomfortable [70].

Iontophoresis is essential in addressing the issues of an aging population and diabetic people with health issues and a lack of access to healthcare thanks to these benefits. While the chemical makeup of workout sweat is significant for uses in physiology and athletics, chemical iontophoresis-generated sweat is more beneficial for medical diagnostics [73,74]. Due to the quick sampling times and the ability to take measurements while sitting, which is more convenient than exercising [75]. Iontophoresis is the best method for extensive population monitoring since it may reduce the risks of exercise-induced hypoglycemia in diabetes individuals. Given that exercise causes the body to go through dynamic physiological changes whereas local sweat stimulation by iontophoresis essentially leaves the body in its resting state.

Iontophoresis, however, might be more appropriate for a single application as opposed to ongoing monitoring, as the repetitive administration of iontophoretic current at the same location could be damaging to the underlying skin. To protect the skin from operational damage and irritation, the wearable sensors should be prepared taking into account the current density and iontophoresis time. As a result of repetitive sweat induction of iontophoresis, the pH of the epidermis will vary due to ionic buildup at the sweat-sampling site. In order to minimize burns brought on by unfavorable pH effects, safeguard the epidermis, and solve this problem, buffered gel coatings may be utilized [71,76]. The Ag nanowire network can be employed as a very transparent and flexible heater for wearable electronics applications because of its better electrical conductivity at high aspect ratios [77]. According to Ko and his coworkers' research, the distinctive structure of the Ag nanowire network/PDMS could provide Joule heating with a quick thermal response, improved electrical stability to endure repeated mechanical stress, and a minimal variation in resistance [78]. Sweat can be produced on the curved and irregular surfaces of the human body thanks to the wearable stretchy heater's soft and thin qualities (e.g., chest, forehead, temple, forearm). The exposed layer of Ag nanowires is coated with gold (Au), a non-toxic, oxidation-resistant material, to produce sweat over an extended period of time utilizing wearable stretchy heaters [77,78]. As a result of the galvanic coating method used on au, the device's reliable, long-term sweat production for continuous sweat glucose monitoring provides improved electrical stability under sweating conditions.

However, if the stretchable wearable heater is made of a soft, thin, solid-layer patch, it will be difficult to wear and will not allow for adequate airflow. The solution to this issue is the use of super hydrophobic materials, which can introduce the sweat into the sensing system and keep the skin dry during the sweating process. This might be a useful method in the field of for non-invasive biosensing of glucose, however it requires power. The use of this method for in-situ sweat glucose monitoring has not been documented in any studies [73].

The market for electrochemical biosensors is being driven by diagnostic and health monitoring applications, notably the growing need for wearable sensors [79,80]. The cost-effective and scalable technology pushed by screen printed electrodes utilized on wearable sensing technology [81] owns the biggest market share of electrochemical systems. In terms of commercial applications, qualities like as adaptability, miniaturization, reliability, and device cost are critical. Several technological difficulties must be considered for the market, including robustness, dependable and fast response, and sensor fouling prevention [82,83]. Furthermore, regulatory agency approval by comparison with standardized analytical techniques is required [84].

MOF-based wearable sensors frequently include a biological component in the sensing matrix, such as enzymes and antibodies, which require special storage conditions. Although specific characteristics of commercially marketed glucometers' excellent storage stability are covered by patent, the capillary chamber where the enzyme is maintained along with mediators and stabilizers appears to play a significant role in it [85]. Despite the commercial success of electrochemical systems, several hurdles remain for applications that rely on long-term stability, as most strips can only be held for two years under optimum conditions [86]. There are several publications and patenting activities connected to the development of electrochemical biosensors for the detection of environmental pollutants, food toxins, and biomarkers, however they are rarely commercialized [87]. The majority of biosensing experiments have been performed under controlled settings and evaluated using buffer solutions deemed "fairly clean" rather than complex real-world materials.

It should be noted that approaches based on enzymatic inhibition can produce false positive results since various substances impede enzyme activity, reducing sensor selectivity and lowering its sensitivity [88]. The restrictions associated with natural enzymes have made the creation of commercially viable biosensors extremely difficult. The principal disadvantages are incompatibility with organic solvents, poor stability, the high expense of demanding extraction and purification operations, and the limited experimental settings of pH, temperature, and ionic strength [89]. The main issue impeding the commercialization of most electrochemical biosensors is their lack of robustness and reliability due to low long-term activity, repeatability, and matrix interference [90]. Another critical issue is enzyme immobilization. Enzymes undergo conformational changes on the electrode surface, leading in activity loss, which decreases biosensor sensitivity and short-term storage [91]. These concerns remain unresolved and necessitate extra efforts. To address the shortcomings of the biological recognition element, artificial enzymes and molecularly imprinted polymers have been developed to mimic enzymatic function or to improve particular binding affinity towards target analytes [92]. These elements provide exceptional opportunities for the development of new biomimetic electrochemical sensors.

Non-enzymatic biosensors based on biomimetic materials are the subject of significant research because they have the potential to overcome recurring stability difficulties, paving the path for mass production and commercialization of viable sensors [93]. In addition, advances in material science and micro-engineering have accelerated the development of wearable, simple-to-use, low-cost, and noninvasive biosensors. They are, however, largely employed for research, and the integration of biomimetic materials with commercial products is still in the works. Furthermore, biofouling is a significant challenge in market applications of these biomimetic materials incorporated into electrochemical sensing platforms [94]. Many diverse species may adsorb on the electrode surface, reducing sensor repeatability, because the electrodes are in close contact with complex matrices to perform the intended analysis. Biofouling is not just a challenge for biomimetic electrochemical sensors; enzymatic-based sensors face the same issue, necessitating ongoing attempts to solve it.

One of the major technological challenges is the gap between the innovative concepts brought by academic research and their integration with market needs, as well as the inaccessibility of biological samples, known as real-world samples, and limited experience on marketable devices. Collaboration between academies and companies could yield a viable solution to the need for more durable, stable, and low-cost sensors.

Table 3 highlights the advantages and disadvantages of MOFs, and provide a comparison of MOFs with other porous nanomaterials.

**Table 3:** Advantageous and disadvantages of MOFs and other nanomaterials.

Porous Materials	Composition	Advantages	Disadvantages	Reference
MOFs	Metal–organic framework (MOF) Organic ligands and their coordinated metal ions/ion clusters	Ordered porous structure, biocompatibility, and ease of functional modification.	Targeting and potential biotoxicity.	[95,96]
COFs	Light elements (H, C, N, O, B)	Large surface area, high thermal stability, good biocompatibility, and good biodegradability.	The synthesis condition is not mild enough, the preparation cost is high, and the structure is uncontrollable.	[97,98]
MIPs	Conductive polymers	Good biocompatibility and excellent selectivity	usually higher effort required for template cleavage after MIP synthesis	[99,100]
LDH	Metals and hydroxide molecules separated by exchangeable anions and water molecules	High conductivity	Complicated and expensive synthesis procedures	[101,102]
CNTs	A layer of carbon atoms that are bonded together in a hexagonal (honeycomb) mesh	High surface area Antifouling	High cost synthesis and purification procedures	[103,104]
Graphene	Carbon atoms positioned in a hexagonal design	Excellent flexibility	Sophisticated synthesis procedures and may be toxic (e.g the use of hydrazine)	[105,106]
Mesoporous silica nanoparticles (MSN)	Silica (SiO2)	Huge loading capacity, controllable particle size and shape, suitability for easy functionalization, and biocompatibility.	Poor dispersibility and stability, prone to accumulation, and requires modification. A fully reversible lid is required to close the pore access.	[107,108]

## 5 Limitations

The prospects presented by MOFs-based wearable sensors are limited in certain ways by several obstacles that have yet to be fully resolved. Ionic strength, temperature, and liquid-junction potentials can all be adjusted for or taken into consideration [109]. Leaching of membrane components, i.e., biocompatibility/toxicity, can be reduced by using membranes based on polymers [110] or perfluorocarbons; such membranes may increase membrane stability and hence the sensor's long-term usefulness.

The use of ordinary addition to overcome the matrix effect is incompatible with the wearable concept and will almost certainly increase the cost of the sensing platform for environmental analysis [111]. For wearable applications, sampling challenges, such as sweat evaporation [112], contribute to a delay in the sensor's commercialization. Finally, the practical implementation of wearable chemical sensors and deployed autonomous systems for environmental trace analysis necessitates significant efforts from mechanical and electronic engineers to solve other issues, such as passive pumping, sensor integration in fabric, microfluidic design, and wireless data transmission [113]. It should be also noted that the miniaturization of the MOFs-based sensors may raise the costs of production and may cause lower potential stability because of the smaller redox or double-layer capacitances, the leakage of membrane components, the adherence and the exfoliation of membranes and overall, a trade-off in decreasing the size of the electrode may exist [114]. It should also be noted that miniaturization of MOFs-based sensors may increase production costs and result in lower potential stability due to smaller redox or double-layer capacitances, membrane component leakage, membrane adherence and exfoliation [115], and overall, a trade-off in decreasing the size of the electrode may exist.

It appears that there aren't any convincing designs for inexpensive, mass-producible ion-selective microelectrodes that can be used for wearable electronics and environmental monitoring. Out-sensor computing consumes a lot of energy and adds a lot of latency. It is critical to create physical intelligent devices that allow near-sensor or in-sensor computing in order to solve this problem. Such a problem might be resolved by machine learning, which might also enhance the utilization of artificial e-skins with multiple wearable sensing nodes for gathering external information. On the other hand, a significant issue for everyday monitoring is the service life of wearable electromechanical sensors. Based on their self-power supply, chemical stability, wearing comfort, and mechanical durability, wearable electromechanical sensors will have an effect on long-term use. Self-repairing hydrogels and elastomers may open new avenues to overcome wearable sensors stability in the future. A unique wearable sensor needs to be built through material design and structural optimization in order to enable long-term monitoring and high-fidelity electrophysiological recording.

In conclusion, there is still a lot of work to be done in order to create wearable sensors based on MOFs that are affordable and dependable for research on untreated real samples.

## 6 Challenges and directions

### 6.1 Challenges

MOFs-based Wearable sensors are characterized by various challenges including usual sensing challenges such as pretreatment, stability and reproducibility, sensitivity challenges and real sample sensitivity along with MOFs wearability related challenges such as materials related challenges, Self-destroyed sensor-based challenges and consumers’ behavior toward such devices. These are also the major challenges in the fabrication of electrochemical biosensors.

1 Stability of MOFs-based wearable sensors is a major issue since they are relied on a biorecognition system that requires a stable environment for the biorecognition element such as enzymes especially for long-term storage.
2 The sensitivity, selectivity and LOD of wearable sensors are the key features of any wearable sensor and ideal biosensors must have a very low value of LOD and outstanding selectivity toward the studied analyte so that they can provide accurate data with negligible hysteresis at lowest interference.
3 Up-scaling wearable technology always confront reproducibility related challenges. To ensure accurate fabrication and marketing of MOFs-based wearable sensors they must have excellent reproducibility and repeatability, so similar sensors with similar features could be produced.
4 To be consider for human health diagnosis, MOF-based wearable sensor must be able to operate properly in a real sample such as saliva, blood, urine, sweat, body fluid, tears, etc. The real sample collection is itself a challenge; some factors need to be considered for collecting a real sample for detection.
5 Limitations due to MOFs morphology, safety and biodegradability for constructing highly performed wearable sensors are one of the most critical issues in developing MOFs-based wearable sensors for health diagnosis. MOFs-based sensors must be safe and have mechanical features so they can be compatible with the studied area (e.g. skin) without interfering the results causing no damage to the patient.
6 MOFs-based wearable sensors must be removed from the body once their mission is accomplished to avoid any side effects which require additional sophisticated procedures. Some health complications may appear if one of the component of the wearable sensor failed to be removed and/or in case of an in-vivo damage which may lead to serious in-vivo injuries during the diagnosis of a disease. Wearable sensors must be resistant to any mechanical and physicochemical changes to avoid any in-vivo issues.
7 In case of commercialization and scaling-up, production costs and customer behavior toward MOFs-based wearable sensor could be considered as a serious challenge. To get a rapid and efficient market diffusion, the customer needs to be convinced about the advantages of the product taking into account its cost, ease of operation with minimal efforts and product safety.
8 Several sensors require calibration before every use and incubation in conditioning solution. Such conditions are incompatible with wearable technology. Furthermore, many forms of sensors have the issue of drift that causes significant error in calculating analyte concentration. Therefore, these sensors need to be re-calibrated at regular intervals. Researchers are addressing this issue by developing calibration-free sensors

### 6.2 Solutions

1 The issue related to conformal contact between wearable electrochemical sensors and the human body can be resolved by matching the mechanical properties of the device with that of the human tissues. The human tissue is characterized as soft, stretchable and curvilinear with unique mechanical properties. In this regard, researcher must conduct more efforts in developing MOFs with outstanding mechanical properties including but not limited to curvilinearity, softness, flexibility and outstanding stretchability.
2 A combination of MOFs and silicon based materials could help in improving MOFs-based wearable sensor toward extreme mechanical and physichochemical changes such as pressure and temperature. Such innovative strategies must be explored by researchers to realize highly biocompatible and biodegradable chemical sensors that are eventually consumed by the body.
3 MOFs functionalization and their combinations with other materials such as graphene, reduced graphene oxide (rGO), carbon nanotubes (CNTs), metals and metal oxide nanoparticles may improve the stability of the biorecognition matrix and then, ensure long-term storage.
4 Developing cost-effective and eco-friendly synthesis procedures to prepare MOFs is highly recommended to ensure safe, biodegradable and cheap wearable sensors.
5 Fast response and Micro-nano MOFs-based wearable sensors can be disposed and removed easily and may improve their performance would involve fewer efforts.
6 The gap between research laboratory and hospitals should be bridged so more trails will conducted and MOFs-based wearable sensors can be tested in real-life circumstances, which will help in evaluating their performance and convincing people to use such tools to diagnosis their health.
7 To ensure up-scaling and excellent market diffusion of such sensors, more efforts should be conducted focusing on the key element for a successful up-scaling strategy including but not limited to production costs, stability and advertising.
8 MOFs-based wearable sensors should benefit from machine learning and artificial intelligence to overcome the aforementioned challenges. Such technologies may offer excellent opportunities to improve sensors analytical performance and improving their stability, controlling and miniaturizing their size.

## 7 Conclusion and future prospects

MOF based wearable sensors showed promising performance for future applications in human health care applications and disease diagnosis. However, these sensors must be sensitive, selective and stable to maintain long term performance. The design and architecture of the MOF-based sensing platform while being doped with other materials such as carbon based materials and metals nanoparticules which have high flexibility, stability, conductivity and trustworthy durability can be useful in the fabrication of high performance MOF-based wearable sensors. The development of high performance MOF-based sensors depends ongoing research and technological advances. Therefore, future researchers will likely focus on the following areas, as stated below.

• Designing eco-friendly and more conductive MOF-based composites to create more stable and sensitive MOF-based sensing platforms that ensure the accuracy and linearity of the produced MOF-based wearable sensors.
• To expand their applications in wearable sensing, MOFs-based sensing platform should be stretchable and flexible which require more efforts on improving their mechanical properties and their biocompatibility taking into account their costs of production.
• To increase wearing comfort and skin affinity, MOF wearable sensors should be created using bionic structures rather than artificial glue.
• Integration of 3D technologies in the fabrication of MF-based wearable sensors since such technologies helps in improving the long term stability and the reproducibility of the sensors, and may also help in the up-calling of these sensors.
• The operational conditions of wearable sensors should be optimized to produce more adaptable MOF-based wearable sensors for each application and to meet a variety of end-user biological needs.
• To produce more accurate and smart MOF-based wearable sensors, machine learning and artificial intelligence should be integrated during the process of production.

## Data availability statement

All data generated or analyzed during this study are included in this published article.

## Funding statement

The publication of this article was funded by the Qatar National Library (QNL). The authors would like to acknowledge the library for supporting the publication of this article.

## CRediT authorship contribution statement

**Hicham Meskher:** Conceptualization, Data curation, Formal analysis, Investigation, Methodology, Project administration, Resources, Writing – original draft, Writing – review & editing. **Samir Brahim Belhaouari:** Funding acquisition, Project administration, Resources, Supervision, Validation, Writing – review & editing. **Fariborz Sharifianjazi:** Methodology, Project administration, Resources, Supervision, Visualization, Writing – review & editing.

## Declaration of competing interest

The authors declare that they have no known competing financialinterestsor personal relationships that could have appeared to influence the work reported in this paper.
